# Sources of Error in the Determination of Sugar in the Urine

**Published:** 1859-01

**Authors:** 


*Sources of Error in the Determination of Sugar in the Urine.*—It has been shown by Leconte, Bonnet and Berlin, that the potass-copper liquid employed in the investigation of the presence of glucose, may undergo, on the part of uric acid, a reduction analagous to that effected by the glucose itself. Babo and Meissner have lately shown that, in such reduction, allantoine is formed, a part of which is converted into urea and oxalic acid.

The coloring matter of the urine is without action on the reagent in question, as is the case with creatine, creatinine and hippuric acid. It is certain, however, that a reduction may be effected by the volatile organic acids.

A constant cause of the reduction has just been discovered by Brucke, which is glucose itself, this substance always being present in normal urine. The author insists on this opinion, since it is not based upon the nature of the products of decomposition, but upon the production of a direct compound of the sugar with potassa—a compound already employed by Lehmann in his examination of sugar in the blood. The process for demonstrating the presence of sugar in normal urine demands great care, and is as follows: Absolute alcohol should be added to the urine so that it may constitute four-fifths of the whole volume. The author of this process ordinarily operates with 200 centilitres, although, with care, 50 centilitres would answer. By the addition of the alcohol the mixture becomes turbid, and a precipitate is produced that can be separated by means of a filter. To the clear liquid an alcoholic solution of potassa is then added, constant stirring being used, until red litmus paper is distinctly blued by the mixture; after which it is allowed to rest for twenty-four hours in a cool place. At the end of this time the liquid is decanted with care, and the glass is reversed on *papier buvard;* when the latter becomes dry, the glass is placed mouth upwards, and suffered to remain until no more alcoholic odor is exhaled; then it will be found that the bottom of the vessel and a portion of the sides will be covered with a crystalline layer, which represents the saccharate of potassa in question. The deposite is rich when it gives the glass a frosted appearance; on the contrary, a clotty or granulated crystillization indicates the presence of foreign matters.

Saccharate of potassa is very soluble in water; and with such a solution it is easy to perform the different experiments necessary for the determiation of glucose—experiments too well known to require their mention here.—*Jour. de Pharm. et de Chemie.* L. H. S.

